# Role of the CX3C chemokine receptor CX3CR1 in the pathogenesis of atherosclerosis after aortic transplantation

**DOI:** 10.1371/journal.pone.0170644

**Published:** 2017-02-24

**Authors:** Zuzanna Rowinska, Thomas A. Koeppel, Maryam Sanati, Hubert Schelzig, Joachim Jankowski, Christian Weber, Alma Zernecke, Elisa A. Liehn

**Affiliations:** 1 Department of Vascular Surgery and Interdisciplinary Vein Center, St. Josef-Hospital, Ruhr- University Bochum, Bochum, Germany; 2 Institute of Molecular Cardiovascular Research, University Hospital, RWTH Aachen University, Aachen, Germany; 3 Division of Vascular Surgery, Hospital Asklepios St. Georg Hamburg, Hamburg, Germany; 4 Department of Vascular and Endovascular Surgery, Düsseldorf University Hospital, Düsseldorf, Germany; 5 School for Cardiovascular Diseases (CARIM), University of Maastricht, Maastricht, The Netherlands; 6 Institut for Prevention and Epidemiology of Cardiovascular Disease, Ludwig-Maximilian-University of Munich, Munich, Germany; 7 Institute of Experimental Biomedicine, University Hospital Würzburg, Würzburg, Germany; 8 Human Genetic Laboratory, University for Medicine and Pharmacy, Craiova, Romania; Klinikum Region Hannover GmbH, GERMANY

## Abstract

**Background:**

The CX3C chemokine receptor CX3CR1 is expressed on monocytes as well as tissue resident cells, such as smooth muscle cells (SMCs). Its role in atherosclerotic tissue remodeling of the aorta after transplantation has not been investigated.

**Methods:**

We here have orthotopically transplanted infrarenal *Cx3cr*1^-/-^*Apoe*^-/-^ and *Cx3cr*1^+/+^*Apoe*^-/-^ aortic segments into *Apoe*^-/-^mice, as well as *Apoe*^-/-^ aortic segments into *Cx3cr*1^-/-^*Apoe*^-/-^ mice. The intimal plaque size and cellular plaque composition of the transplanted aortic segment were analyzed after four weeks of atherogenic diet.

**Results:**

Transplantation of *Cx3cr*^-/-^*Apoe*^-/-^ aortic segments into *Apoe*^-/-^ mice resulted in reduced atherosclerotic plaque formation compared to plaque size in *Apoe*^-/-^ or *Cx3cr*1^-/-^*Apoe*^-/-^ mice after transplantation of *Apoe*^-/-^ aortas. This reduction in lesion formation was associated with reduced numbers of lesional SMCs but not macrophages within the transplanted *Cx3cr*^-/-^ *Apoe*^-/-^ aortic segment. No differences in frequencies of proliferating and apoptotic cells could be observed.

**Conclusion:**

These results indicate that CX3CR1 on resident vessel wall cells plays a key role in atherosclerotic plaque formation in transplanted aortic grafts. Targeting of vascular CX3CL1/CX3CR1 may therefore be explored as a therapeutic option in vascular transplantation procedures.

## Introduction

Atherosclerosis is an inflammatory disease of the arteries with a high rate of morbidity and mortality in the Western World. The immune reactions involved in atherosclerotic plaque formation are controlled by lipid mediators, costimulatory molecules and cytokines. Chemokines are a family of chemotactic cytokines that mediate immune cell recruitment and are of high relevance in atherogenesis [1], and targeting of chemokines and their receptors has been shown to reduce experimental atherosclerosis [2–4].

Fractalkine or CX3CL1 is a CX3C chemokine that is expressed in the vessel wall and mediates firm adhesion and chemotaxis of leukocytes expressing its G-protein-coupled receptor, CX3CR1. CX3CL1 is the only member of the CX3C chemokine family and is usually attached to the membrane through a mucin-like domain [5,6].

Fractalkine/CX3CL1 expression has been detected on activated endothelial cells, smooth muscle cells (SMCs), macrophages and platelets, and is involved in the development of numerous inflammatory pathologies including atherosclerosis, rheumatoid arthritis, or graft rejection [7–13]. While the expression of fractalkine and its receptor CX3CR1 is low in healthy vessels, it increases significantly under pathological conditions, such as in atherosclerotic plaques or during allograft rejection [11,13]. In atherosclerotic vessels, its receptor CX3CR1 is expressed on monocytes and tissue resident SMCs. Mice deficient in CX3CR1 (*Cx3cr*^-/-^ mice) have previously been employed to investigate the function of monocyte survival and recruitment during atherogenesis [14,15]. The CX3CL1-CX3CR1 interaction has been shown to confer essential survival signals in monocytes/macrohages, and absence of CX3CR1 was shown to lead to an increased death of monocytes and/or foam cells, inhibiting atherosclerotic lesion formation [15]. Pharmacological inhibition of the chemokine receptor, CX3CR1 has furthermore been described to inhibit leukocyte recruitment and to reduce atherosclerosis [16], indicating an important role of CX3CL1 in the pathogenesis of atherosclerosis. In addiiton, platelets express CX3CR1, which plays a role in the formation of platelet-monocyte complexes [10]. Moreover, CX3CL1/Fractalkine can elicit chemotactic responses in SMCs and has been described to exert anti-apoptotic and proliferative effects on human vascular SMC [6,11].

Effects of a deficiency of CX3CL1/CX3CR1 or its blockade *in vivo*, however, do not allow a discriminatation of local from systemic effects. In this regard, murine aortic transplant models have attracted great attention as these allow studying specific vascular responses caused by the graft itself separate from systemic factors related to the recipient (atherogenic) environment [17–19].

In this study, we focused on the function of CX3CR1 in the atherosclerotic response within an aortic segment graft orthotopically transplanted in mice. This allowed us to investigate the role of vascular CX3CR1 versus systemic CX3CR1 to gain further insight into the details of how this chemokine receptor controls atherosclerotic plaque formation.

## Methods

### Aortic transplantation

*Apoe*^-/-^ mice (C57BL/6 background) were crossed with *Cx3cr1*^-/-^ [20] mice to generate *Cx3cr1*^+/+^ *Apoe*^-/-^ and *Cx3cr1*^-/-^ *Apoe*^-/-^ mice. 8–12 week old male mice were housed under standardized conditions in the Animal Facility of the University Hospital Aachen (Germany). The operating procedure was in accordance with European legislation and approved by local German authorities. Infrarenal abdominal *Apoe*^-/-^ aorta segments were transplanted into *Apoe*^-/-^ mice (n = 4), *Cx3cr1*^-/-^ *Apoe*^-/-^ aorta segments into *Apoe*^-/-^ mice (n = 5), and *Apoe*^-/-^ aorta segements were transplanted into *Cx3cr1*^-/-^*Apoe*^-/-^ mice (n = 5). After transplantation, mice were placed on atherogenic diet (21% fat, 0.15% cholesterol, Altromin) for four weeks.

The abdominal aortic transplantation was performed as described previously using the sleeve technique [17,21]. Briefly, mice were anesthetized using a mixture of 1.5 vol. % isoflurane and 100% oxygen through a face mask. The segment of aorta between the renal arteries and its bifurcation was separated from the vena cava. The infrarenal aorta of the recipient was dissected and mobilized between the renal arteries proximally and the bifurcation distally. All the small branches of this segment were secured. The proximal and distal portions of the aorta were clamped by 6/0 single silk suture (Silk, Deknatel, Research Triangle Park NC, USA). The graft was placed in the orthotopic position and the anastomosis was performed using the sleeve technique with sutures 11/0 monofilament (Ethilon, Ethicon, Norderstedt, Germany). The mean surgical anastomosis time was 22 minutes (range 13–40 minutes). Donor and recipient mice were of the same age (8–12 weeks).

### Immunohistochemistry

For tissue harvesting, animals were anesthetized (with a mixture of 1.5 vol. % isoflurane and 100% oxygen via a face mask) and vessels were flushed with phosphate-buffered saline (PBS) followed by 4% formaldehyde/PBS (pH 7.4) by cardiac puncture. Grafts were gently removed. After overnight fixation in 4% formaldehyde/PBS, specimens were further processed and embedded in paraffin. Serial sections (5 μm) throughout the transplanted aorta distal and proximal of the anastomosis sites were stained by hematoxylin and eosin staining (HE). Immunocytochemistry was performed using antibodies raised against macrophages (Mac2, CL8942AP/Cedarlane), vascular smooth muscle cells (SMA, M0851/Dako) followed by anti-rat FITC (Jackson Immuno- Research, West Grove, USA) and anti-mouse-Cy3, respectively, antigen KI-67 for cellular proliferation (KI-67, M7249/Dako, Jena, Germany) and TUNEL for detecting DNA fragmentation (1215679210/Roche; 11684795910/Roche, Mannheim, Germany). Positively stained cells were counted in higher magnification (40x) and expressed as percent from total DAPI stained nuclei or as absolute numbers per mm^2^ (S1A and S1B Fig).

### Statistics

Data are presented as mean ± SEM. The significance of changes between experimental groups and different time points was determined by one-way ANOVA, with Tukey multiple comparison test. A p-value of <0.05 was considered significant. Statistical analyses were performed with GraphPad Prism 6.03 (Statcon, Witzenhausen, Germany).

## Results

Murine orthotopical transplantations of infrarenal aortic segments of *Apoe*^-/-^ mice into *Apoe*^*-/-*^ mice (control, n = 4), of *Cx3cr1*^*-/-*^ *Apoe*^-/-^ aortic segments into *Apoe*^-/-^ mice (n = 5), as well as *Apoe*^-/-^ aortic segments into *Cx3cr1*^-/-^ *Apoe*^-/-^ mice (n = 5) were performed using the sleeve technique [17,21], and mice were placed on atherogenic diet containing 21% fat and 0.15% cholesterol. Four weeks after transplantation, the aortic segments were harvested for further analysis.

**Fig 1:**
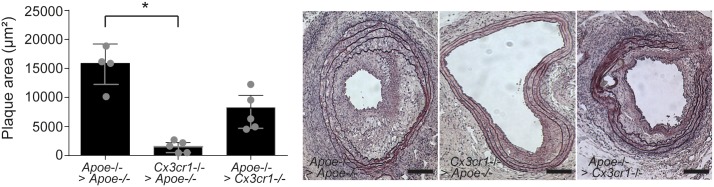
Reduced plaque size after transplantation of Cx3cr1^-/-^Apoe^-/-^ aortas into Apoe^-/-^ mice. *Apoe*^-/-^ mice were transplanted with *Apoe*^-/-^ aortic segments (n = 4, *Apoe*^-/-^ > *Apoe*^-/-^) or *Cx3cr*1^-/-^*Apoe*^-/-^ aortic segments (n = 5, *Cx3cr*1^-/-^ > *Apoe*^-/-^), and *Cx3cr*1^-/-^*Apoe*^-/-^ mice were transplanted with *Apoe*^-/-^ aortic segments (n = 5, *Apoe*^-/-^ > *Cx3cr*1^-/-^) and placed on a high fat diet for 4 weeks. Atherosclerotic plaques were analysed in H&E stained sections through the transplanted segment. Quantification of plaque area and representative sections (x20) are shown. *p<0.05, Scale bars 100 μm.

The histological analysis of the transplanted aortic segment revealed a significant reduction in plaque size in *Cx3cr1*^*-/-*^ *Apoe*^-/-^ aortic segments in *Apoe*^-/-^ recipients, whereas a trend towards reduced plaque burden was observed in *Apoe*^-/-^ aortic segments transplanted into *cx3cr1*^*-/-*^ *Apoe*^-/-^ mice did not reach statistical significance, compared to control *Apoe*^-/-^ aortic segments in *Apoe*^-/-^ mice (Fig 1). The histological analysis of the transplanted aortic segment revealed a significant reduction in plaque size in *Cx3cr1*^*-/-*^ *Apoe*^-/-^ aortic segments in *Apoe*^-/-^ recipients, whereas a trend towards reduced plaque burden was observed in *Apoe*^-/-^ aortic segments transplanted into *Cx3cr1*^*-/-*^ *Apoe*^-/-^ mice did not reach statistical significance, compared to control *Apoe*^-/-^ aortic segments in *Apoe*^-/-^ mice (Fig 1). No statistical differences in plaque size could be detected between *Cx3cr1*^*+/+*^ *Apoe*^-/-^ mice with *Cx3cr1*^*-/-*^ *Apoe*^-/-^ aortic segments and *Cx3cr1*^*-/-*^ *Apoe*^-/-^ mice with *Cx3cr1*^*+/+*^ *Apoe*^-/-^ aortic segments.

**Fig 2:**
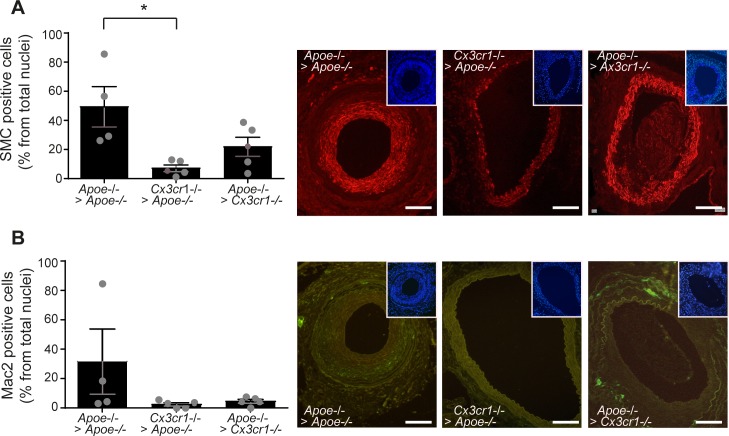
Reduced numbers of SMCs in transplanted Cx3cr1^-/-^Apoe^-/-^ aortic plaques. *Apoe*^-/-^ mice were transplanted with *Apoe*^-/-^ aortic segments (n = 4, *Apoe*^-/-^ > *Apoe*^-/-^) or *Cx3cr1*^*-/-*^*Apoe*^-/-^ aortic segments (n = 5, *Cx3cr1*^*-/-*^ > *Apoe*^-/-^), and *Cx3cr1*^*-/-*^*Apoe*^-/-^ mice were transplanted with *Apoe*^-/-^ aortic segments (n = 5, *Apoe*^-/-^ > *cx3cr*1^-/-^) and placed on a high fat diet for 4 weeks. Immunohistochemistry and quantification of (A) SMCs (alpha-Smooth Muscle Actin staining, x20, red) and (B) macrophages (MAC2, x20, green) in plaques of the transplanted aortic segment. Counterstaining with DAPI (blue) is showed in each images in insert. *p<0.05, Scale bars 100 μm.

Immunostaining further revealed that this decrease in atherosclerotic plaque size in *Cx3cr1*^-/-^ *Apoe*^-/-^ aortic segments was associated with a significant reduction in the number of smooth muscle actin (SMA)^+^ SMCs in plaques of the aortic segment (Fig 2A, S1C Fig). The presence of SMA^+^ SMCs mostly in the intima during plaque formation may indicate that SMCs in the media differentiate towards a synthetic phenotype during vessel inflammation and plaque formation [22]. In contrast, lesional Mac2^+^ macrophage numbers were not significantly altered between groups (Fig 2B, S1D Fig). These findings suggest that CX3CR1 expressed by vessel wall-resident SMCs is required for atherosclerotic plaques growth in transplanted vessels, whereas its expression on circulating cells and macrophages seems to play a subordinate role.

Furthermore, we assessed cell proliferation by Ki-67 staining in all plaque cells and apoptosis by TUNEL staining in all plaque cells but could not detect significant differences between groups (S1E and S1F Fig).

## Discussion

We here employed a model of orthotopic transplantation of an infrarenal aortic segment into the infrarenal aorta of recicpient mice. Unlike in bone marrow transplantation models, which can be used to dissect the contribution of bone marrow versus non-bone marrow cells (including vessel wall cells but also all resident cells in other organs), this model allows to focus on the particular contribution of local (transferred) cells within the graft. Using this aorta transplantation model to discriminate between effects of circulating versus local vessel wall resident cells, we here are able to show that interference with the CX3CR1-driven signaling pathway in vessel wall cells inhibits atherosclerotic lesion formation.

Previous studies by Combadière *et al*. and Lesnik *et al*. demonstrated a significant decrease in atherosclerotic lesions in mice lacking CX3CR1 [23,24], associated with a reduction in macrophage accumulation in plaques [23,24]. Saederup *et al*. showed that the deletion of CX3CL1 in CCR2-deficient mice reduced the accumulation of macrophages and the development of atherosclerosis [25]. In accordance, Combadière *et al*. demonstrated that the combined inhibition of CCL2, CX3CR1, and CCR5 in *Apoe*^-/-^ mice was associated with a marked and additive reduction in atherosclerosis [26]. The site-specific action of CX3CR1 was not addressed in these studies. However, also bone marrow deficiency of CX3CR1 reduced atherosclerotic lesion formation in chimeric *Apoe*^-/-^ mice, and the CX3CL1/CX3CR1 signaling pathway was uncovered to exert pro-survival signals in macrophages, whose absence leads to the increased death of monocytes/foam cells during plaque growth [15]. In contrast to these findings, we did not observe significant alterations in plaque size or lesional macrophage accumulation in *Cx3cr1*^-/-^ *Apoe*^-/-^ mice transplanted with *Apoe*^-/-^ mice that entail a deficiency in CX3CR1 in circulating leukocytes. This may be due to specificities of the transplantation model that produces plaques that predominantly contain SMCs rather than macrophages in the infrarenal abdominal aorta.

The interaction of SMCs with monocytes induces a significant up-regulation of CX3CR1 gene and protein expression in both cell types [27]. Moreover, CX3CL1 gene and protein expression increases in the murine aorta in high-fat *vs* normal diet fed *Apoe*^-/-^ mice [28]. Notably, CX3CL1 has been demonstrated to exert anti-apoptotic and proliferative effects on vascular cells, mediated via EGFR, with antagonists of CX3CR1 abrogating the proliferative effects of CX3CL1 in SMCs [6]. Deficiency of CX3CR1 may similarly have curtailed CX3CL1-mediated SMC proliferation, in turn inhibiting intimal hyperplasia. However, we cannot rule out the involvement of other vessel wall cells, given that we did not observe changes in SMC or proliferating cells relative to the plaque area. For instance, resident myeloid cells that accumulate CX3R1-dependently in the vessel wall [29] may have contributed to the phenotype observed.

Clinically, stability of an atherosclerotic plaque is an important characteristic [30]. Unstable atherosclerotic plaques are characterized by high numbers of macrophages and decreased SMC and collagen content. These plaques are more likely to undergo rupture and cause vessel thrombosis when compared to SMC and collagen-rich stable plaques [30]. Our findings that a decrease in atherosclerotic plaque size in *Cx3cr1*^-/-^ *Apoe*^-/-^ aortic segments was associated lower numbers of SMCs may indicate that inhibition of CX3CR1 may also have unfavorable effects on plaque stability. On the other hand, confining SMC proliferation is major challenge in vascular remodeling after percutanous transluminal coronary angioplasty and stent-implantation [31]. In this regard, targeting CX3CR1 may be a potent therapeutic target limiting monocyte accumulation [15,23,24] and SMC proliferation.

An increased expression of CX3CL1 and CX3CR1 has been found in coronary arteries from patients after heart transplantation, and several studies have suggested a role of CX3CR1 in allograft rejection and dysfunction [13,32–34]. For instance, Robinson *et al*. observed a prolonged *in vivo* survival of heterotopic murine cardiac allograft transplants in a major histocompatibility complex-mismatch model, when treated with CX3CR1-antibodies [13]. In our study, however, both donors and recipients were of the C57BL/6 background, excluding the possibility that (CX3CR1-dependent) graft versus host responses may have affected atherosclerotic lesion development in our transplant model.

A clear limitation of our study are the low n-numbers that may have precluded reaching more definite statistical conclusions. In particular, the trend of reduced plaque burden in Apoe-/- aortic segments transplanted into Cx3cr1-/- Apoe-/- mice compared to control Apoe-/- aortic segments in Apoe-/- mice may have substantiated when group sized were lager. Similarly, the cellular plaque characterization yielded values with a wide distribution, so that significant differences between groups may have been missed. Moreover, an additional group of mice with *Cx3cr1*^-/-^ donors and *Cx3cr1*^-/-^ recipients is missing to evaluate the complete absence of Cx3cr1 and possible additive effects in our model. Taken together we here unveil an important function of CX3CR1 in resident vessel wall cells to promote atherosclerotic lesion growth in the transplanted abdominal aorta in *Apoe*^-/-^ mice. Targeting CX3CL1/CX3CR1 may thus be viable therapeutic option in vascular transplantation procedures.

## Supporting information

S1 Fig Assesment of cell content by immunofluorescence. (A) The positive stained sections for smooth muscle actin (SMA in red) was overlay with DAPI and the nuclei inside the stained area were counted (red arrow heads). Scale bar 50 μm. (B) Similar, nuclei inside the Mac2-stained area (green) are counted (green arrow heads). Immunohistochemistry and quantification of (C) SMCs and (D) macrophages in plaques of the transplanted aortic segment and expressed as cells/mm^2^. (E) No change is detected after staining with Ki-67 for proliferation and (F) TUNEL for apoptosis.
(TIF)

S1 Table The original raw data table. The original raw data table (in a Excel spreadsheet) that were used to generate our bar graphs.
(XLSX)

## References

[1] Charo IF, Ransohoff RM. The many roles of chemokines and chemokine receptors in inflammation. N Engl J Med. 2006; 354: 610–621. 10.1056/NEJMra052723 16467548

[2] Back M, Weber C, Lutgens E. Regulation of atherosclerotic plaque inflammation. J Internal Med. 2015; 278: 462–482. 10.1111/joim.12367 25823439

[3] Zernecke A, Shagdarsuren E, Weber C. Chemokines in atherosclerosis: an update. Arterioscler Thromb Vasc Biol. 2008; 28:1897–1908. 10.1161/ATVBAHA.107.161174 18566299

[4] Liehn EA, Cabrera-Fuentes HA. Inflammation between defense and disease: impact on tissue repair and chronic sickness. Discoveries. 2015; 3: e42. 10.15190/d.2015.34 PMC6941570 32309565

[5] Fong AM, Robinson LA, Steeber DA, Tedder TF, Yoshie O, Imai T, et al Fractalkine and CX3CR1 mediate a novel mechanism of leukocyte capture, firm adhesion, and activation under physiologic flow. J Exp Med. 1998; 188: 1413–1419. 9782118 10.1084/jem.188.8.1413 PMC2213407

[6] White GE, Tan TC, John AE, Whatling C, McPheat WL, Greaves DR. Fractalkine has anti-apoptotic and proliferative effects on human vascular smooth muscle cells via epidermal growth factor receptor signalling. Cardiovasc Res. 2010; 85: 825–835. 10.1093/cvr/cvp341 19840952 PMC2819832

[7] Bazan JF, Bacon KB, Hardiman G, Wang W, Soo K, Rossi D, et al A new class of membrane-bound chemokine with a CX3C motif. Nature. 1997; 385: 640–644. 10.1038/385640a0 9024663

[8] Ludwig A, Berkhout T, Moores K, Groot P, Chapman G. Fractalkine is expressed by smooth muscle cells in response to IFN-gamma and TNF-alpha and is modulated by metalloproteinase activity. J Immunol. 2002; 168: 604–612. 11777952 10.4049/jimmunol.168.2.604

[9] Greaves DR, Häkkinen T, Lucas AD, Liddiard K, Jones E, Quinn CM, et al Linked chromosome 16q13 chemokines, macrophage-derived chemokine, fractalkine, and thymus- and activation-regulated chemokine, are expressed in human atherosclerotic lesions. Arterioscler Thromb Vasc Biol. 2001; 21: 923–929. 11397698 10.1161/01.atv.21.6.923

[10] Postea O, Vasina EM, Cauwenberghs S, Projahn D, Liehn EA, Lievens D, et al Contribution of platelet CX(3)CR1 to platelet-monocyte complex formation and vascular recruitment during hyperlipidemia. Arterioscler Thromb Vasc Biol. 2012; 32: 1186–1193. 10.1161/ATVBAHA.111.243485 22383701

[11] Lucas AD, Bursill C, Guzik TJ, Sadowski J, Channon KM, Greaves DR. Smooth muscle cells in human atherosclerotic plaques express the fractalkine receptor CX3CR1 and undergo chemotaxis to the CX3C chemokine fractalkine (CX3CL1). Circulation. 2003; 108: 2498–2504. 10.1161/01.CIR.0000097119.57756.EF 14581400

[12] Ruth JH, Volin MV, Haines GK, Woodruff DC, Katschke KJ Jr, Woods JM, et al Fractalkine, a novel chemokine in rheumatoid arthritis and in rat adjuvant-induced arthritis. Arthritis Rheum. 2001; 44: 1568–1581. 10.1002/1529-0131(200107)44:7<1568::AID-ART280>3.0.CO;2-1 11465708

[13] Robinson LA, Nataraj C, Thomas DW, Howell DN, Griffiths R, Bautch V, et al A role for fractalkine and its receptor (CX3CR1) in cardiac allograft rejection. J Immunol. 2000; 165: 6067–6072. 11086038 10.4049/jimmunol.165.11.6067

[14] Apostolakis S, Spandidos D. Chemokines and atherosclerosis: focus on the CX3CL1/CX3CR1 pathway. Acta Pharmacol Sin. 2013; 34: 1251–1256. 10.1038/aps.2013.92 23974513 PMC4002164

[15] Landsman L, Bar-On L, Zernecke A, Kim KW, Krauthgamer R, Shagdarsuren E, et al CX3CR1 is required for monocyte homeostasis and atherogenesis by promoting cell survival. Blood. 2009; 113: 963–972. 10.1182/blood-2008-07-170787 18971423

[16] Poupel L, Boissonnas A, Hermand P, Dorgham K, Guyon E, Auvynet C, et al Pharmacological inhibition of the chemokine receptor, CX3CR1, reduces atherosclerosis in mice. Arterioscler Thromb Vasc Biol. 2013; 33: 2297–2305. 10.1161/ATVBAHA.112.300930 23887641

[17] Dambrin C, Calise D, Pieraggi MT, Thiers JC, Thomsen M. Orthotopic aortic transplantation in mice: a new model of allograft arteriosclerosis. J Heart Lung Transplant. 1999; 18: 946–951. 10561104 10.1016/s1053-2498(99)00051-0

[18] Daugherty A. Mouse models of atherosclerosis. Am J Med Sci. 2002; 323: 3–10. 11814139 10.1097/00000441-200201000-00002

[19] Xu Q. Mouse models of arteriosclerosis: from arterial injuries to vascular grafts. Am J Pathol. 2004; 165: 1–10. 10.1016/S0002-9440(10)63270-1 15215157 PMC2216680

[20] Jung S, Aliberti J, Graemmel P, Sunshine MJ, Kreutzberg GW, Sher A, et al Analysis of fractalkine receptor CX(3)CR1 function by targeted deletion and green fluorescent protein reporter gene insertion. Mol Cell Biol. 2000; 20: 4106–4114. 10805752 10.1128/mcb.20.11.4106-4114.2000 PMC85780

[21] Rowinska Z, Zander S, Zernecke A, Jacobs M, Langer S, Weber C, et al Non- invasive in vivo analysis of a murine aortic graft using high resolution ultrasound microimaging. Eur J Radiol. 2012; 81: 244–249. 10.1016/j.ejrad.2010.12.083 21334152

[22] Swier VJ, Tang L, Krueger KD, Radwan MM, Del Core MG, Agrawal DK. Coronary Injury Score Correlates with Proliferating Cells and Alpha-Smooth Muscle Actin Expression in Stented Porcine Coronary Arteries. PlosOne, 2015; 10(9): e0138539. 10.1371/journal.pone.0138539 PMC4575201 26382957

[23] Combadière C, Potteaux S, Gao JL, Esposito B, Casanova S, Lee EJ, et al Decreased atherosclerotic lesion formation in CX3CR1/apolipoprotein E double knockout mice. Circulation. 2003; 107: 1009–1016. 12600915 10.1161/01.cir.0000057548.68243.42

[24] Lesnik P, Haskell CA, Charo IF. Decreased atherosclerosis in CX3CR1-/- mice reveals a role for fractalkine in atherogenesis. J Clin Invest. 2003; 111: 333–340. 10.1172/JCI15555 12569158 PMC151849

[25] Saederup N, Chan L, Lira SA, Charo IF. Fractalkine deficiency markedly reduces macrophage accumulation and atherosclerotic lesion formation in CCR2-/- mice: evidence for independent chemokine functions in atherogenesis. Circulation. 2008; 117: 1642–1648. 10.1161/CIRCULATIONAHA.107.743872 18165355 PMC3589525

[26] Combadière C, Potteaux S, Rodero M, Simon T, Pezard A, Esposito B, et al Combined inhibition of CCL2, CX3CR1, and CCR5 abrogates Ly6C(hi) and Ly6C(lo) monocytosis and almost abolishes atherosclerosis in hypercholesterolemic mice. Circulation. 2008; 117: 1649–1657. 10.1161/CIRCULATIONAHA.107.745091 18347211

[27] Butoi ED, Gan AM, Manduteanu I, Stan D, Calin M, Pirvulescu M, et al Cross talk between smooth muscle cells and monocytes/activated monocytes via CX3CL1/CX3CR1 axis augments expression of pro-atherogenic molecules. Biochim Bioph Acta. 2011; 1813: 2026–2035. 10.1016/j.bbamcr.2011.08.009 21888931

[28] Liu H, Jiang D, Zhang S, Ou B. Aspirin inhibits fractalkine expression in atherosclerotic plaques and reduces atherosclerosis in ApoE gene knockout mice. Cardiovasc Drugs Ther. 2010; 24: 17–24. 10.1007/s10557-009-6210-7 19997773

[29] Liu P, Yu YR, Spencer JA, Johnson AE, Vallanat CT, Fong AM, et al CX3CR1 deficiency impairs dendritic cell accumulation in arterial intima and reduces atherosclerotic burden. Arterioscler Thromb Vasc Biol. 2008; 28: 243–250. 10.1161/ATVBAHA.107.158675 18079406

[30] Virmani R, Kolodgie FD, Burke AP, Farb A, Schwartz SM. Lessons from sudden coronary death: a comprehensive morphological classification scheme for atherosclerotic lesions. Arterioscler Thromb Vasc Biol. 2000; 20: 1262–1275. 10807742 10.1161/01.atv.20.5.1262

[31] Lenzen MJ, Boersma E, Bertrand ME, Maier W, Moris C, Piscione F, et al Management and outcome of patients with established coronary artery disease: the Euro Heart Survey on coronary revascularization. Eur Heart J. 2005; 26: 1169–1179. 10.1093/eurheartj/ehi238 15802360

[32] Hoffmann U, Bergler T, Segerer S, Rümmele P, Krüger B, Banas MC, et al Impact of chemokine receptor CX3CR1 in human renal allograft rejection. Transpl Immunol. 2010; 23: 204–208. 10.1016/j.trim.2010.06.006 20600902

[33] Cao G, Lu Y, Gao R, Xin Y, Teng D, Wang J, et al Expression of fractalkine, CX3CR1, and vascular endothelial growth factor in human chronic renal allograft rejection. Transplant Proc. 2006; 38: 1998–2000. 10.1016/j.transproceed.2006.06.081 16979977

[34] Hu H, Aizenstein BD, Puchalski A, Burmania JA, Hamawy MM, Knechtle SJ. Elevation of CXCR3-binding chemokines in urine indicates acute renal-allograft dysfunction. Am J Transplant. 2004; 4: 432–437. 14961998 10.1111/j.1600-6143.2004.00354.x

