# Supplementary figures and images for: Role of the CX3C chemokine receptor CX3CR1 in the pathogenesis of atherosclerosis after aortic transplantation
Source: PLoS One. 2017 Feb 24;12(2):e0170644. doi: 10.1371/journal.pone.0170644 (PMC5325192; doi:10.1371/journal.pone.0170644)

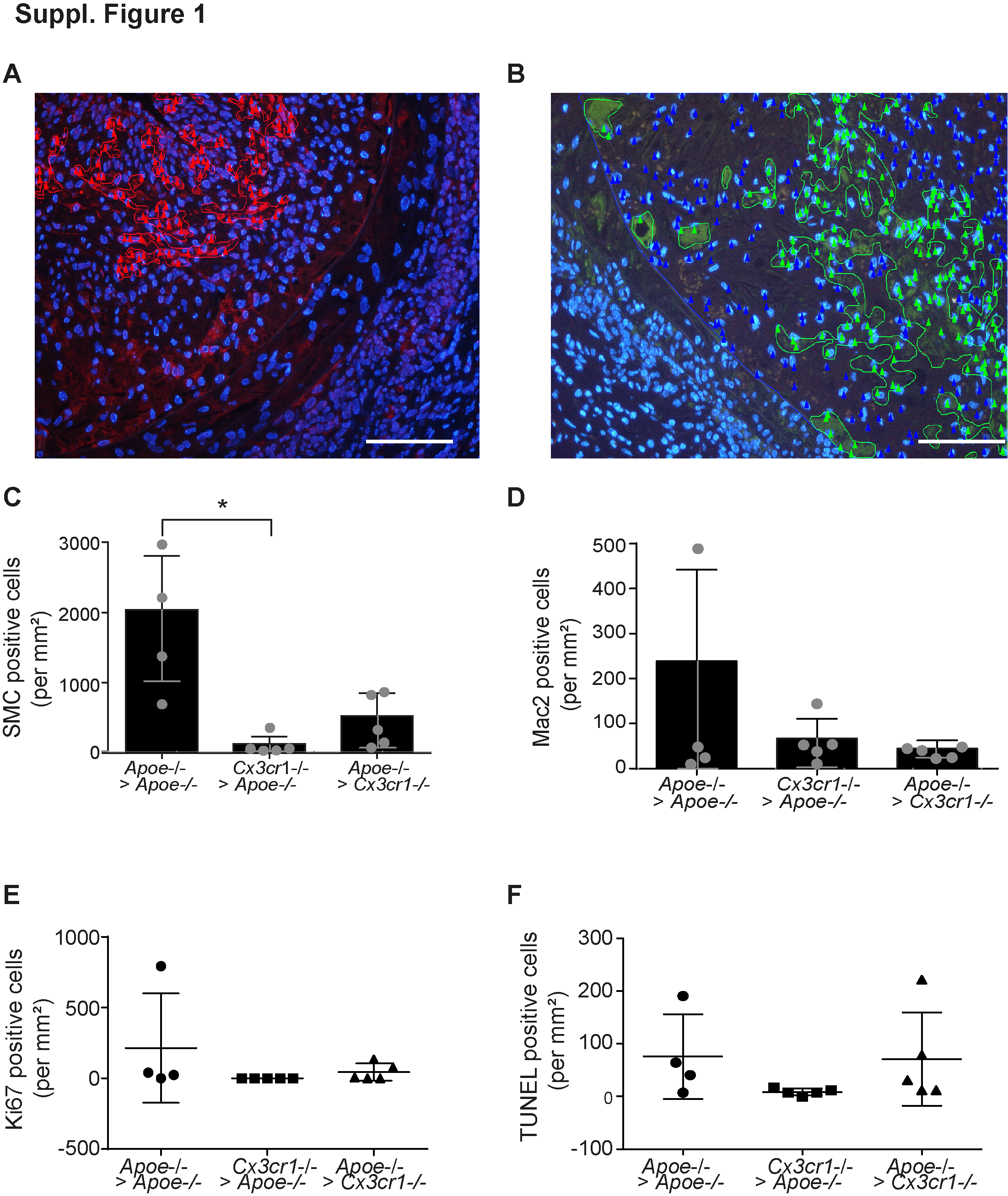

Supplement: S1 Fig — (A) The positive stained sections for smooth muscle actin (SMA in red) was overlay with DAPI and the nuclei inside the stained area were counted (red arrow heads). Scale bar 50 μm. (B) Similar, nuclei inside the Mac2-stained area (green) are counted (green arrow heads). Immunohistochemistry and quantification of (C) SMCs and (D) macrophages in plaques of the transplanted aortic segment and expressed as cells/mm2. (E) No change is detected after staining with Ki-67 for proliferation and (F) TUNEL for apoptosis. (TIF) [file pone.0170644.s001.tif]
